# 

**Published:** 1910-04-15

**Authors:** 


### ANNOUNCEMENTS.

To the Dental Profession of America. At the December 1909 meeting of the Ohio State Dental Society it was unanimously resolved that an American Memorial be established to perpetuate the memory of the late Dr. Willoughby D. Miller, as an evidence of the profession's appreciation of his laborious and fruitful researches for the scientific advance of dentistry.

From the concensus of opinion of various State and local societies it was decided that the memorial take the form of a monument, to be erected in a suitable public place in Columbus, Ohio, the capital of Miller's native State. The monument to consist of a life size bronze of Dr. Miller mounted upon a granite base of suitable proportions with appropriate tablets, the cost of which will approximate $8,000.

Though his scientific career was in a foreign land, the great pride he showed in his American citizenship, the love for his profession in America, and his final plans for educating students in his own country in the line of work he had so ably begun, should make this memorial movement National in its scope, and to this end the committee in charge has selected Honorary Committees in the several states to co-operate in bringing this matter to a successful issue.

This movement has received the endorsement of the National Dental Association. Ohio will raise $1,200 for this fund and it is the desire of the committee to have one tablet to state that contributions were received from representatives of the profession in every state of the Union.

Contributions are desired from individuals as well as societies, in fact, many small subscriptions are preferable to a few large ones.

The committee has selected Dr. Weston A. Price, 10406 Euclid Avenue, Cleveland, Ohio, to act as treasurer of this fund, and to him all subscriptions should be made payable.

That your State may be represented in this fund and appear in the published list of subscribers, we ask your earnest support. Your response to this appeal will be the measure, not only of the success of our committee but of the appreciation of American dentists for one who raised the standard of the profession.

Yours very respectively,
EDWARD C. MILLS, *Chairman*,
16 South Third St., Columbus, Ohio.
J. R. CALLAHAN,
25 Garfield Place, Cincinnati, Ohio.
S. R. RUGGLES,
Portsmouth, Ohio.

MICHIGAN STATE DENTAL SOCIETY. "The next Annual meeting of the Michigan State Dental Society will be held in Detroit June 13th, 14th and 15th, 1910"

THE FORTIETH ANNUAL MEETING of the New Jersey State Dental Society, will convene in Asbury Park, N. J., at the "New Casino" July 20th, 21, 22nd, and 23rd, 1910.

This is to be the largest meeting ever held, two days will be devoted to difficult and interesting clinics which should not be missed. Many fine and instructive papers to be. read. Hotel Columbia will be the headquarters of the society.

Asbury Park is ideal for the meetings of the society, with its ocean for bathing and many commodious hotels. The Casino is on the beach front, large and cool. The entire floor of the Casino is to be leveled for the exhibits which will be many and varied and everything new will be shown. Asbury Park can be readily reached by rail, boat or motor and will be a good resort for the visitors to bring their families who will find much entertainment of high class while they attend the meetings. No one should miss this meeting. Prospective members, ethical practitioners of other states and members of local societies are cordially welcome. For further information inquire of the secretary,

CHARLES A. MEEKER, D.D.S.,
29 Fulton St., Newark, N. J.

THE OHIO STATE DENTAL EXAMINER.—Will hold an examination in Columbus June 21 to 24, 1910.

DR. L. L. YONKER, *Secy.*
Bowling Green, O.

THE IOWA STATE BOARD.—Will hold an examination in Iowa City, June 6, 1910.

DR. E. D. BOWERS, *Secy.*

Le Mars.

THE MICHIGAN BOARD OF EXAMINERS.—Will hold an examination in Ann Arbor, June 20, 1910.

DR. A. W. HAIDLE, *Secy.*
Negaunee, Mich.

TENNESSEE STATE DENTAL ASSOCIATION.—Meets in Nashville, May 17 to 19, 1910.

